# Cytomegalovirus colitis unmasking human immunodeficiency virus infection as a cause of IgA vasculitis

**DOI:** 10.1186/s12981-023-00545-9

**Published:** 2023-07-19

**Authors:** Alice Bartoletti, Paolo Delvino, Marco Minetto, Alessandra Milanesi, Emanuele Bozzalla Cassione, Verdiana Serena Quadrelli, Ombretta Luinetti, Sara Monti, Carlomaurizio Montecucco

**Affiliations:** 1 Department of Internal Medicine and Therapeutics, Università di Pavia, Pavia, Italy; 2 Division of Rheumatology, Fondazione IRCCS Policlinico San Matteo, Piazzale Golgi 2, 27100 Pavia, Italy; 3 Experimental Medicine, University of Pavia, Pavia, Italy; 4 Department of Molecular Medicine, Unit of Anatomic Pathology, University of Pavia, Fondazione IRCCS Policlinico San Matteo, Pavia, Italy

**Keywords:** HIV, AIDS, IgA vasculitis, CMV

## Abstract

**Background:**

Human immunodeficiency virus (HIV) has a protean clinical picture, in rare instances manifesting as systemic autoimmune disorders such as vasculitides. HIV-induced autoimmune diseases often do not respond well to systemic immunosuppressive therapy. Opportunistic infections may occur in patients with either acquired immunodeficiency syndrome (AIDS) or heavy immunosuppressive treatment, and can further complicate the clinical presentation.

**Case presentation:**

A patient presenting with immunoglobulin A (IgA) vasculitis (IgAV) with treatment-refractory purpuric skin rash and suspect intestinal vasculitis was discovered to have AIDS. HIV was the trigger of IgAV, and cytomegalovirus (CMV) colitis mimicked intestinal vasculitis. Antiretroviral treatment improved both CMV colitis and the control of the autoimmune disease.

**Conclusions:**

An autoimmune disease relapsing despite adequate immunosuppressive treatment and/or the presence of recurrent severe opportunistic infections may be clues to an underlying HIV infection.

## Background

Human immunodeficiency virus (HIV) is a rare and frequently neglected cause of autoimmune rheumatic diseases, including vasculitides, and may complicate their clinical course [1]. The presence of unusual features like an autoimmune condition resistant to the conventional immunosuppressive treatment alongside recurrent severe opportunistic infections may hinder the diagnosis of an underlying HIV infection.

We hereby describe a case of occult long-standing HIV infection that caused treatment-refractory immunoglobulin A (IgA) vasculitis (IgAV) and cytomegalovirus (CMV) colitis, the latter mimicking intestinal vasculitis.

## Case presentation

**Fig. 1:**
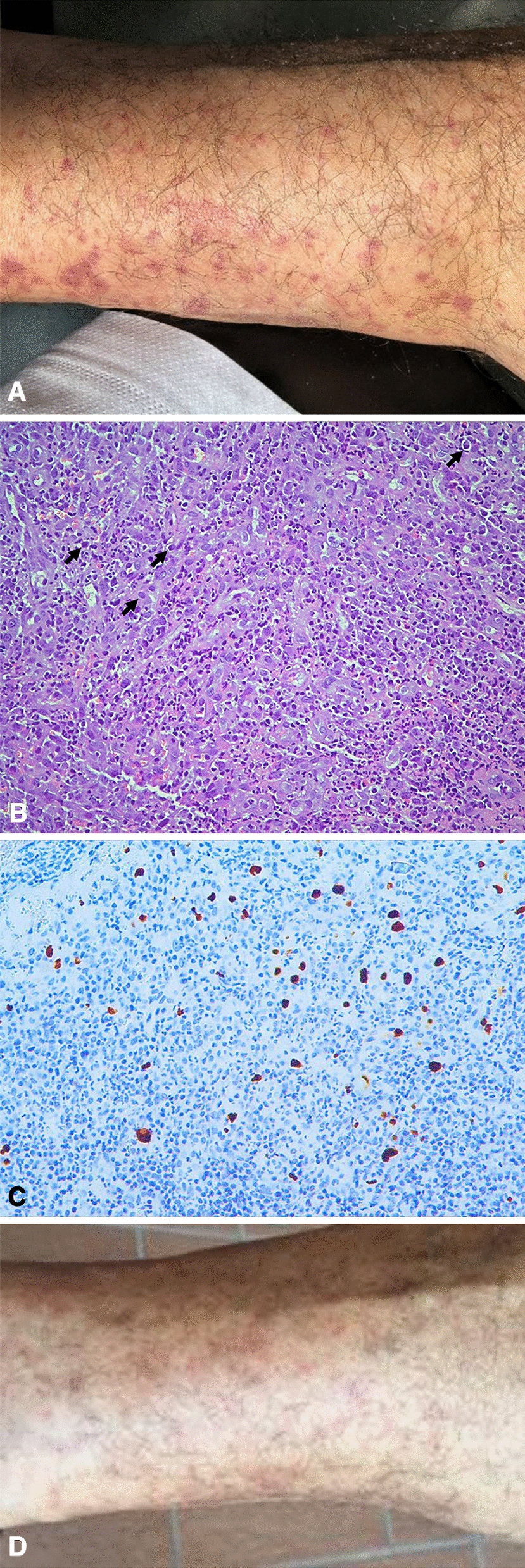
**A** Purpuric lesions of the lower limbs at disease presentation. **B** Histology of caecal mucosa (Haematoxylin and Eosin): granulation tissue with endothelial hyperplasia, widespread infiltration of eosinophils, histiocytes and macrophages, intranuclear Owl’s eye inclusion bodies consistent with CMV infection (arrows). **C** CMV immunohistochemistry on caecal mucosa: positive reaction within the nuclei of CMV-infected cells. **D** Purpuric lesions of the lower limbs after treatment

A 65-year-old Caucasian male came to our attention with a 4-month history of purpuric lesions of the lower limbs (Fig. 1A), arthralgias, low-grade fever, weight loss, abdominal pain, and nausea. The patient denied any ear-nose-throat, lung, neurological or genitourinary symptom. Petechiae extended up to the buttocks, they were palpable, slightly pruritic, sub-centimetric in diameter and did not blanch when pressure was applied. His health records were unremarkable apart from systemic hypertension and Epstein-Barr virus (EBV) infection two years earlier. Routine laboratory tests showed normochromic normocytic anaemia, white blood cell count 6250/µL (neutrophils 3390/µL, lymphocytes 1540/µL, eosinophils 540/µL), erythrocyte sedimentation rate 40 mm/h, C-reactive protein 1.6 mg/dL, normal kidney function and urinalysis, and pronounced polyclonal hypergammaglobulinemia with raised IgA (660 mg/dL, ULN 500 mg/dL) and IgG (2790 mg/dL, ULN 2530 mg/dL). The autoimmune panel detected ANA 1:160 cytoplasmic fibrillar pattern (AC-15,16,17), with negative anti-ENA, anti-dsDNA, rheumatoid factor, anti-neutrophil cytoplasmic antibodies and cryoglobulins; complement was not consumed. The patient was negative for hepatitis B and C infection. Serum and urine immunofixation were negative, and the haematology consultant ruled out an ongoing lymphoproliferative disease.

IgAV was suspected, thus further studies aimed at investigating vasculitis were performed. Punch skin biopsy showed leukocytoclastic vasculitis and direct immunofluorescence detected IgA deposits within small vessels of the dermis. Urine sediment analysis did not reveal any sign of active glomerulopathy. Colonoscopy found a caecum ulcer of 25 mm in diameter. A diagnosis of IgAV with cutaneous and suspected gastrointestinal involvement was confirmed, hence the patient started prednisone 50 mg/day with disappearance of arthralgias and abdominal pain, with partial resolution of petechiae. Upon glucocorticoid tapering, he experienced a recrudescence of cutaneous pruritic rash involving the trunk and lower limbs. Prednisone was increased to 37.5 mg/day and azathioprine 100 mg/day was added with moderate improvement of skin lesions.

Meanwhile, he developed perianal vesicular lesions positive for herpes simplex virus 1 and 2, and the histological examination of the intestinal ulcer revealed intranuclear CMV inclusion bodies (Fig. 1B, C). Following these findings, blood tests for EBV-DNA and CMV-DNA were performed, resulting in 102,420 copies/mL and 2997 copies/mL respectively. Considering the multiple infections and the clinical picture not completely responding to high-dose glucocorticoids, HIV infection was suspected. HIV antibody and p24 antigen resulted positive, with a viral load of 122,791 copies/mL and 12 CD4^+^ T lymphocytes/µL. The patient was diagnosed with acquired immunodeficiency syndrome (AIDS), disseminated CMV infection with CMV colitis, EBV reactivation, and cutaneous IgAV. He started dolutegravir/lamivudine and ganciclovir together with prophylaxis for opportunistic infections, whereas azathioprine was interrupted, and glucocorticoids slowly tapered. After antiviral treatment initiation, viral loads progressively decreased and the CD4^+^ T cell count rose slightly. Nonetheless, the patient experienced a flare of petechial lesions on the lower limbs one month after glucocorticoid discontinuation. Colchicine was started with prompt improvement of the skin rash (Fig. 1D).

## Discussion

IgAV is a systemic condition characterised by the deposition of IgA-containing immunocomplexes within small vessels inducing inflammation, blood leakage and subsequent organ damage. Skin, joints, gastrointestinal system and kidneys are the most commonly involved districts [2]. IgAV can be idiopathic, but sometimes an infectious process, either bacterial, viral or parasitic, may be the trigger [1].

We described a case of cutaneous IgAV that was difficult to manage with conventional immunosuppressants, along with unexplained polyclonal hypergammaglobulinemia, mild eosinophilia and a concomitant opportunistic infection. These rather unusual features led us to suspect an underlying cause to the vasculitis. Actively replicating HIV infection was the primordial trigger of IgAV, hence immunosuppressive treatment alone was insufficient to achieve disease remission. HIV infection portends immune system deregulation, thus increasing the risk of secondary autoimmune diseases [3]. The reported prevalence of autoimmune disorders among HIV patients is 6–9%, mostly occurring at about 10 years from the time of infection [4, 5]. Among these, vasculitides are < 1% and they are more commonly found before highly active antiretroviral therapy implementation and at rather low CD4^+^ T lymphocyte counts [4, 6, 7]. Some opportunistic pathogens can possibly contribute themselves to vasculitis development, especially among AIDS patients with CD4^+^  < 200/µL [4, 6]. Interestingly, in our case an opportunistic infection was responsible for an intestinal lesion imitating vasculitis.

Treatment of HIV-related autoimmune diseases first consists of antiretroviral therapy optimisation. If symptoms are not controlled, immunosuppressants can be added [8]. However, such treatment can pose an additional threat, due to an increased risk of opportunistic infections.

To sum up, while facing treatment-resistant rheumatic diseases, the differential diagnosis is crucial to uncover potential triggers or mimickers of autoimmune disorders, such as infections or neoplasms. Similarly, when opportunistic infections appear in otherwise healthy patients or at relatively mild degrees of immunosuppression, an underlying condition should be sought after.

## Data Availability

Data sharing is not applicable to this article as no datasets were generated or analysed during the current study.

## Abbreviations

AIDS: Acquired immunodeficiency syndrome
ANA: Anti-nuclear antibody
CMV: Cytomegalovirus
dsDNA: Double-stranded DNA
EBV: Epstein-Barr virus
ENA: Extractable nuclear antigens
HIV: Human immunodeficiency virus
IgAV: Immunoglobulin A vasculitis
ULN: Upper limit normal
